# Proton Pump Inhibitors Enhance the Antitumor Effect of Chemotherapy for Esophageal Squamous Cell Carcinoma

**DOI:** 10.3390/cancers14102395

**Published:** 2022-05-12

**Authors:** Shinya Matsumura, Takeshi Ishikawa, Juichiro Yoshida, Ryuichi Morita, Tomoki Sakakida, Yuki Endo, Toshifumi Doi, Ryohei Hirose, Ken Inoue, Osamu Dohi, Naohisa Yoshida, Kazuhiko Uchiyama, Tomohisa Takagi, Hideyuki Konishi, Kohichiroh Yasui, Yuji Naito, Yoshito Itoh

**Affiliations:** 1 Department of Molecular Gastroenterology and Hepatology, Graduate School of Medical Science, Kyoto Prefectural University of Medicine, Kyoto 602-8566, Japan; matsumu@koto.kpu-m.ac.jp (S.M.); jyoshida@koto.kpu-m.ac.jp (J.Y.); mryuich@koto.kpu-m.ac.jp (R.M.); stomoki@koto.kpu-m.ac.jp (T.S.); endo0622@koto.kpu-m.ac.jp (Y.E.); t-doi@koto.kpu-m.ac.jp (T.D.); ryo-hiro@koto.kpu-m.ac.jp (R.H.); keninoue71@koto.kpu-m.ac.jp (K.I.); osamu-d@koto.kpu-m.ac.jp (O.D.); naohisa@koto.kpu-m.ac.jp (N.Y.); k-uchi@koto.kpu-m.ac.jp (K.U.); takatomo@koto.kpu-m.ac.jp (T.T.); hkonishi@koto.kpu-m.ac.jp (H.K.); ynaito@koto.kpu-m.ac.jp (Y.N.); yitoh@koto.kpu-m.ac.jp (Y.I.); 2 Department of Gastroenterology, Kyoto Chubu Medical Center, Kyoto 629-0197, Japan; 3 Department of Nursing, Faculty of Health Sciences, Bukkyo University, Kyoto 603-8301, Japan; yasuik@koto.kpu-m.ac.jp

**Keywords:** esophageal cancer, vacuolar ATPase (V-ATPase), proton pump inhibitors (PPIs), 5-Fluorouracil (5-FU), lansoprazole, esomeprazole, vonoprazan

## Abstract

**Simple Summary:**

The use of proton pump inhibitors (PPIs) as V-ATPase inhibitors has been reported to enhance the effectiveness of chemotherapy in some cancers. This study aimed to evaluate the effect of PPIs on 5-Fuorouracil (5-FU)-based therapy for advanced esophageal cancer based on in vitro experiments and a clinical study. In the present study, PPIs showed a dose-dependent antitumor effect on esophageal cancer cells and enhanced the sensitivity of esophageal cancer cells to 5-FU at sublethal concentrations. In the clinical setting, patients treated with oral PPIs showed a superior tumor response to 5-FU and better overall survival in comparison to the non-PPI group. These results indicate that PPIs can enhance chemosensitivity in esophageal cancer patients treated with 5-FU.

**Abstract:**

Background: Vacuolar ATPase (V-ATPase) is involved in cancer development. The use of proton pump inhibitors (PPIs) as V-ATPase inhibitors has been reported to enhance the effectiveness of chemotherapy in certain cancers. This study aimed to evaluate the effect of PPIs on chemotherapy for esophageal cancer. Methods: To investigate the effects of PPIs on esophageal cancer cells, human KYSE50 and 70 cells were plated and 3 PPIs (lansoprazole, esomeprazole, vonoprazan) were added at various concentrations with 5-Fluorouracil (5-FU) to the corresponding cells for a cell viability assay. To investigate the effects of PPI treatment on patients undergoing 5-FU-based therapy in the clinical setting, we retrospectively analyzed the clinical outcomes and chemotherapy-related adverse events in 40 esophageal cancer patients who received 5-FU chemotherapy in our hospital between May 2013 and April 2017. Results: In the viability assays, all PPIs significantly enhanced the cytotoxic effect of 5-FU on the two esophageal cancer cell lines. In the clinical study, PPI-treated patients showed better overall survival (OS) than patients managed without PPI treatment. A multivariate analysis revealed that PPI treatment was independently associated with OS (*p* = 0.009, HR, 0.33; 95% CI, 0.12–0.76). Conclusions: PPI treatment may safely enhance chemosensitivity in esophageal cancer patients.

## 1. Introduction

Esophageal cancer causes more than half a million cancer-related deaths worldwide each year, and squamous cell carcinoma is the most prevalent (87.8%), especially in Asia and Eastern Africa, and alcohol and tobacco are important risk factors for that histological subtype [1,2]. Most patients are unresectable or metastatic disease at diagnosis, and many cases treated with curative intent have a relapse. Although 5-Fuorouracil (5-FU)-platinum-based chemotherapy has been a widely used for these cases, it often results in poor outcomes (median survival, <1 year) [3]. One reason for this result is acquired resistance to anticancer drugs; thus, overcoming acquired resistance is essential for improving the survival of advanced/metastatic cases treated with chemotherapy [4].

The upregulation of glycolysis (the Warburg effect), a universal property of cancers, leads to microenvironmental acidosis, which leads to evolution to phenotypes resistant to acid-induced cell toxicity [5]. Protons produced by glycolysis are transported across the membrane from cancer cells by the overexpressed proton pump, which prevents intracellular acidosis and relieves them from dangerous protons. Among these proton pumps, the most prominent is vacuolar H+-ATPase (V-ATPase) [6]. V-ATPase is an ATP-driven proton pump that functions to both transport protons across the plasma membrane and to acidify intracellular compartments [7]. The action of this pump leads to the selection of more aggressive tumor cell phenotypes that are able to survive in this highly hostile microenvironment. The acidic tumor microenvironment has also been shown to increase the chemoresistance of solid tumors [8]. The expression of V-ATPase has been reported to be associated with pathological grade, TNM stage and tumor metastasis in esophageal squamous cancer cells. The expression of V-ATPase may be strongly associated with drug resistance and tumor metastasis [9]. The extracellular pH of solid tumors is lower than that of normal tissues, which inhibits the uptake of weakly basic chemotherapeutic drugs and, hence, reduces their cytotoxicity [10].

Recently, the inhibition of the acidic microenvironment by blocking the activity of V-ATPases with the use of cytotoxic agents has been shown to synergistically kill chemotherapy-resistant tumors [11,12,13]. Proton pump inhibitors (PPIs), such as lansoprazole and esomeprazole, inhibit gastric acid secretion by targeting the gastric acid pump, and are used to treat gastrointestinal disorders (e.g., gastroesophageal reflux disease, duodenal ulcers and gastric ulcers [14,15,16]), and are commonly used concomitant to cancer treatment. PPIs, which were previously thought to be specific inhibitors of H+/K(+)-ATPase, also inhibit V-ATPase [17,18,19]. Recent studies have demonstrated that the sensitivity of various tumors to chemotherapeutic agents can be enhanced by PPIs [20,21,22]. Thus, PPIs may be a chemosensitizer that enhances the sensitivity of esophageal cancer cells to chemotherapeutic agents. The aim of this study was to evaluate the effect of PPIs on cytotoxicity of 5-Fuorouracil (5-FU) in vitro and the clinical impact of PPIs on the response to chemotherapy for patients with unresectable or recurrent esophageal cancer.

## 2. Materials and Methods

### 2.1. Cell Culture

Human esophageal cancer cells (KYSE50 and KYSE70 cells), obtained from the JCRB Cell Bank (Osaka, Japan), were grown in D-MEM (high glucose) with L-Glutamine and Phenol Red medium supplemented with 10% heat-inactivated fetal bovine serum (FBS) (Thermo Fisher Scientific, Waltham, MA, USA), 100 μg/mL streptomycin and 100 U/mL penicillin at 37 °C under 5% CO_2_ and 90% humidity. The cells were grown in a single cell layer that was attached to treated plastic surfaces, and were subcultured 1 or 2 times per week. In the experiments, cells during the exponential growth phase were used.

### 2.2. Western Blotting

Cellular debris was removed by washing with ice-cold PBS. Then, the cells were lysed in Lysis Buffer (CelLytic M; Sigma-Aldrich; Merck KGaA, Darmstadt, Germany), then scraped and incubated on ice for 15 min. Supernatants were collected, and total protein was mixed with SDS sample buffer. The protein content was measured using the Bradford method; 20 μg of protein was used per lane. The protein lysates were then separated by electrophoresis on 10% SDS-PAGE, then transblotted to polyvinylidene fluoride membranes (Atto Corporation, Tokyo, Japan). Membranes were blocked using 10% EzBlock (Atto Corporation, Tokyo, Japan) in TBS-T (10 mM Tris-HCl (pH 8.0), 150 mM NaCl, 0.1% Tween-20 V/V) for 60 min at room temperature. They were then washed 3 times with TBS-T, and incubated overnight at 4 °C with mouse anti-human-V-ATPase C1 (Santa Cruz Biotechnology, Inc., Dallas, TX, USA) and goat anti-mouse-β-actin (Abcam, Cambridge, UK) antibodies in TBS-T (diluted 1:500–1:1000). Membranes were then washed with TBS-T 3 times, and incubated with secondary anti-mouse (GE Healthcare Japan Corporation, Tokyo, Japan) and anti-goat (GE Healthcare Japan Corporation, Tokyo, Japan) IgG antibodies in TBS-T (diluted 1:5000–1:10,000) for 1 h at room temperature. An ECL-kit (ECL plus, GE Healthcare Bio-Sciences K.K., Tokyo, Japan) was used to detect immunoreactive proteins. Blots were analyzed using ImageJ (version 1.51).

### 2.3. Cell Viability Assay

A Cell Counting Kit-8 (DOJINDO Laboratories, Kumamoto, Japan) was applied to evaluate cell viability. KYSE50 and KYSE70 cells were plated at 1 × 10^4^ cells per well in 100 μL of D-MEM with 10% FBS in 96-well plates for 24 h. Then, 3 types of PPIs (lansoprazole (FUJIFILM Wako Pure Chemical Corporation, Osaka, Japan), esomeprazole (Cayman Chemical Company, Ann Arbor, MI, USA), and vonoprazan (Cayman Chemical Company, Ann Arbor, MI, USA) at various concentrations were freshly prepared and added to the corresponding cells. After 72 h, 10 μL of Cell Counting Kit-8 solution was added to each well, and cells were incubated for another 4 h. The absorbance was measured using a multi-well spectrophotometer (SpectraMax M2, Molecular Devices, San Jose, CA, USA) at 450 nm. Cell viability was calculated as follows: cell viability (%) = absorbance of treated wells/absorbance of control wells (without PPIs) × 100.

In the assay for the combined use of PPIs and 5-FU, various concentrations of 5-FU (2, 20 and 200 μM, FUJIFILM Wako Pure Chemical Corporation, Osaka, Japan) were added to cells 24 h after the administration of a PPI. After another 48 h, we evaluated cell viability. In this case, the sublethal dose was defined and set as the PPI concentration at which >85% of cells of each type were viable at 72 h after PPI administration.

The combination index (CI) was calculated by Compusyn software (ComboSyn, Inc., Paramus, NJ, USA). The quantitative definition of drug combinations is CI = 1 for additive effect, CI < 1 for synergism, and CI > 1 for antagonism [23].

### 2.4. Measurement of Intracellular pH

Intracellular pH (pHi) was measured using the fluorescent pH indicator (2-carboxyethyl)-5-carboxyfluorescein (BCECF)-AM (Molecular Probes, Eugene, OR, USA) according to the manufacturer’s protocol. BCECF-AM was diluted to 1 mM with DMSO. KYSE50 and KYSE70 cells were plated at 1 × 10^4^ cells per well in 100 μL of D-MEM with 10% FBS in a 96-well optical bottom plate polymerBase Black (Thermo Fisher Scientific, Waltham, MA, USA) for 24 h. Then, 3 types of PPIs (lansoprazole, esomeprazole and vonoprazan) at the sublethal concentrations were freshly prepared and added to the corresponding cells. After 72 h, BCECF-AM was diluted to 3 μnol/L with HEPES-buffered Ringer solution (DOJINDO Laboratories, Kumamoto, Japan), adjusted to pH 7.4. Next, the culture medium was replaced, and cells were incubated at 37 °C in a humidified atmosphere containing 5% CO_2_, for 15 min. Then, the culture medium was washed several times with HEPES, and pHi was measured using BCECF fluorescence (excitation wavelength: of 440 nm and 490 nm). Using a multi-well spectrophotometer, at 37 °C, fluorescence intensities were measured every 25 s and monitored at a wavelength of 535 nm. Data acquisition was conducted using SoftMax Pro (v5.4.6, Molecular Devices, San Jose, CA, USA). Calibration of the measurements of each experiment was performed by successively replacing HEPES-buffered Ringer solution with modified Ringer solution at pH 6.4, 6.8, 7.4 and 7.8, with each replacement solution containing the K+/H+ exchanger nigericin at a concentration of 10 μmol/L (Sigma-Aldrich, St. Louis, MO, USA), in order to determine the pHi.

### 2.5. Clinical Research

**Figure 1:**
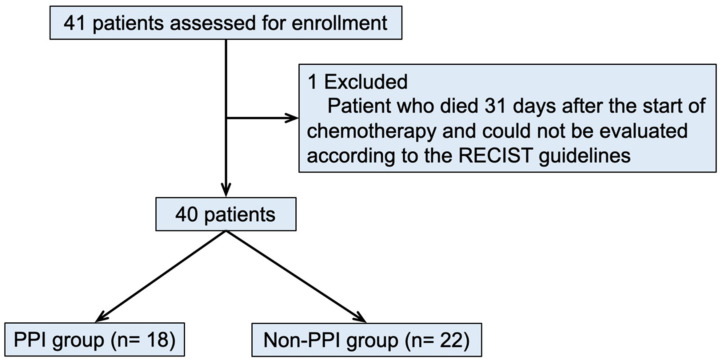
Flow chart of patient enrollment.

We retrospectively investigated the clinical outcomes and chemotherapy-related adverse events of 40 consecutive patients who underwent 5-FU-based chemotherapy for esophageal squamous cell carcinoma (clinical stage IVB according to the 8th edition the Union for International Cancer Control (UICC) staging of cancers of the esophagus) in our hospital between May 2013 and April 2017 (Figure 1) [24]. The 40 cases included patients with postoperative recurrence and patients who received radiotherapy for palliative treatment (e.g., radiation therapy for bone metastases). Patients who used a PPI for at least 30 days from the start of the first chemotherapy treatment were defined as the PPI group (*n* = 18), other patients were defined as the non-PPI group (*n* = 22). The efficacy of treatment was assessed by overall survival (OS) and the response to treatment at the end of 2 courses (RECIST criteria ver. 1.1). The common Terminology Criteria for Adverse Events version 5.0 (CTCAE) was used to assess adverse events. Patient data were collected from their electronic medical records. The present study was approved by the Medical Ethics Review Committee of Kyoto Prefectural University of Medicine (approval no. ERB-E-42). All procedures in this study were in accordance with the ethical standards of the Medical Ethics Review Committee of Kyoto Prefectural University of Medicine, as well as the Declaration of Helsinki. The requirement for informed consent from individual study participants was waived by the Medical Ethics Review Committee of Kyoto Prefectural University of Medicine due to the retrospective nature of this study.

### 2.6. Statistical Analysis

Quantitative data were evaluated using Student’s t-test. A one-way ANOVA followed by post hoc Steel’s multiple comparison test was used to compare multiple groups. Fisher’s exact test was used to compare categorical variables. Overall survival was compared according to the Kaplan–Meier method using a log-rank test. *p* values of <0.05 were considered to indicate statistical significance. JMP Pro (version 14.0, SAS International Inc., Cary, NC, USA) was used to perform the statistical analyses.

## 3. Results

### 3.1. In Vitro Experiment

**Figure 2:**
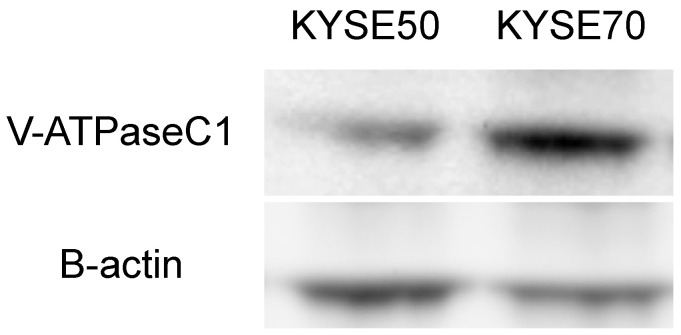
Western blotting to determine for protein expression of V-ATPase in esophageal cancer cell lines.

We evaluated the efficiency of PPIs in increasing the chemosensitivity of the KYSE50 and KYSE70 cell lines to 5-FU. First, we examined the expression of V-ATPase on KYSE50 and KYSE70 cells by western blotting (Figure 2, the whole western blots can be found at Figure S1). We confirmed that V-ATPase was constitutively expressed at the protein level in both cell lines, and was more highly expressed in KYSE70 cells. 

**Figure 3:**
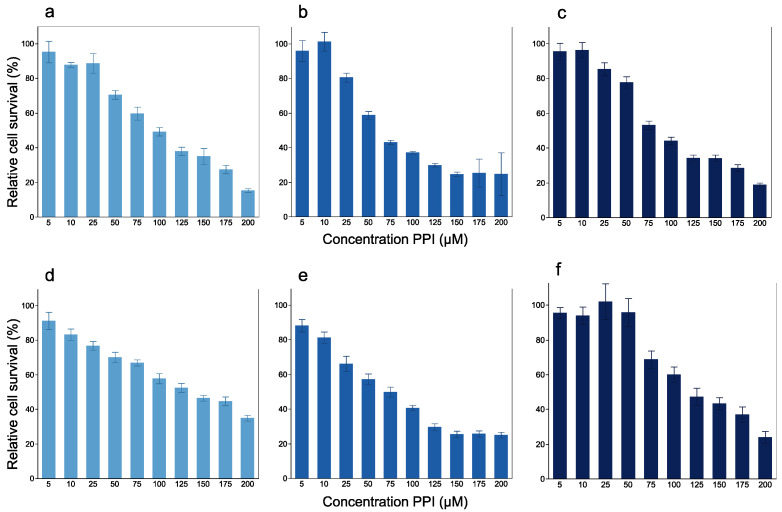
The dose–response bar graph for PPI treatment at various concentrations in the KYSE50 (**a**–**c**) and KYSE70 (**d**–**f**) cell lines. KYSE50 cells treated with (**a**) lansoprazole, (**b**) esomeprazole and (**c**) vonoprazan. KYSE70 cells treated with (**d**) lansoprazole, (**e**) esomeprazole and (**f**) vonoprazan.

Next, we assessed whether PPIs impacted the survival of esophageal cancer cell lines. Figure 3 shows an overview of the dose–response bar graph of PPI treatment at various concentrations. In both cell lines, PPIs reduced cell viability in dose-dependent manner, thus providing evidence to support that PPI treatment had a negative impact on the survival of these cells. In this context, the sublethal doses (defined as a survival rate of at least 85%) were 25 μM for lansoprazole, 10 μM for esomeprazole and 10 μM for vonoprazan in KYSE50 cells, and 5 μM for lansoprazole, 5 μM for esomeprazole and 50 μM for vonoprazan in KYSE70 cells.

**Figure 4:**
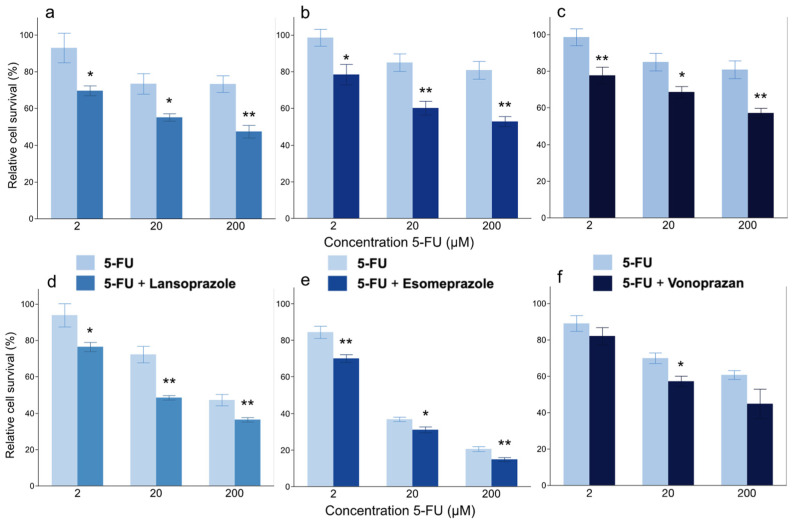
Effect of PPIs on the sensitivity of KYSE50 (**a**–**c**) and KYSE70 (**d**–**f**) cells to 5-FU. KYSE50 cells with (**a**) lansoprazole, (**b**) esomeprazole and (**c**) vonoprazan. KYSE70 cells with (**d**) lansoprazole, (**e**) esomeprazole and (**f**) vonoprazan. *: *p* < 0.05, **: *p* < 0.01.

We investigated whether PPIs at sublethal concentrations increased the sensitivity of both cell lines to 5-FU in vitro. The combination of sublethal concentrations of PPIs with 2, 20 or 200 µM 5-FU reduced the viability of both cell lines at all 5FU concentrations in comparison to cells without PPI treatment (Figure 4). These results suggest that PPIs may increase sensitivity of esophageal cancer to 5-FU.

To test the potential synergistic effects of combination therapy, drug combination index (CI) were analyzed by CompuSyn software (Table S1). All combinations except combination of 2 μM of 5-FU and 50 μM vonoprazane against KYSE70 cells showed a combined synergistic effect (CI < 1).

**Figure 5:**
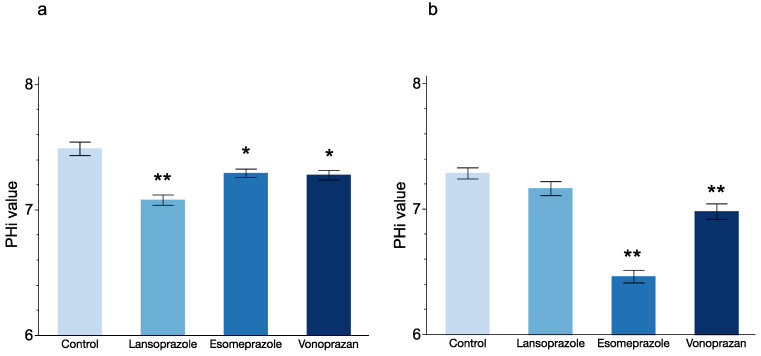
Effect of PPI treatment on intracellular pH. The figure presents the results of intracellular pH measurement of KYSE50 (**a**) and KYSE70 (**b**) cells at 72 h after treatment with sublethal concentrations of PPIs. *: *p* < 0.05, **: *p* < 0.01.

Since, in addition to an acidic extracellular microenvironment, alkaline cytosols have been shown to play a role in resistance to chemotherapy [25], it was considered that a decrease in cytoplasmic pH due to PPI treatment is one of the mechanisms through which PPIs increased the sensitivity of esophageal cancer cell lines to 5-FU. Therefore, intracellular pH (pHi) after PPI treatment was measured using BCECF-AM (a fluorescent pH indicator). The pHi in KYSE50 cells treated with each sublethal concentration of lansoprazole, esomeprazole and vonoprazan for 72 h was significantly lower in comparison to untreated controls (*p* ≤ 0.001, *p* = 0.042 and *p* = 0.017 respectively, Figure 5a). In KYSE70 cells, the pHi was significantly lower at each sublethal concentration of esomeprazole and vonoprazan. (*p* < 0.001 and *p* = 0.004, respectively). The pHi of lansoprazole-treated KYSE70 cells was not significantly different from that of untreated control cells (Figure 5b). 

### 3.2. Clinical Research

The baseline characteristics in a retrospective study that compared two groups (PPI group (*n* = 18) and non-PPI group (*n* = 22)), are shown in Table 1. There were no significant difference in the clinical background factors (e.g., age, sex, body mass index, performance status, the Charlson comorbidity index and selected chemotherapy regimen). The Glasgow prognostic score (GPS), prognostic nutrition index (PNI), and neutrophil/lymphocyte ratio (NLR) at the start of treatment, which are reported to be prognostic factors, were compared; however, there was no significant difference between the two groups. 

According to the RECIST guidelines, the response rate after 2 courses of chemotherapy was 66.7% in the PPI group and 40.9% in the non-PPI group (*p* = 0.102), while the disease control rate was 94.4% and 77.3% (*p* = 0.113), respectively (Table 2), both were better in the PPI group; however, the difference did not reach the statistical significance.

**Figure 6:**
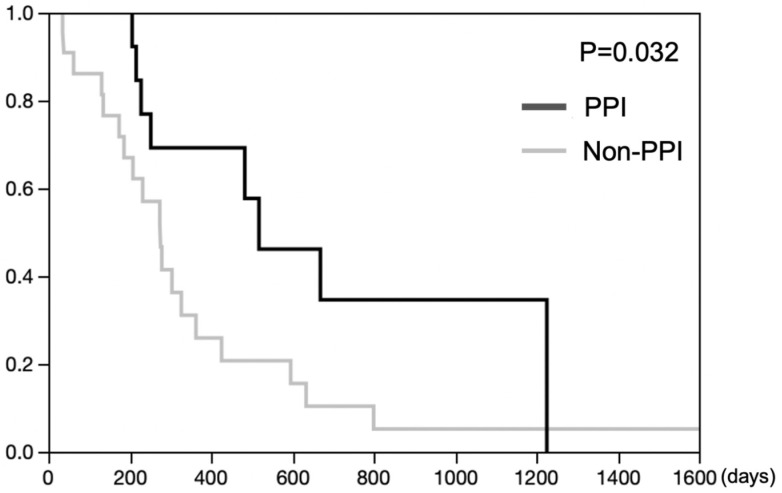
Overall survival (OS) according to PPI use or non-use in esophageal cancer patients receiving 5-FU-based chemotherapy.

The Kaplan–Meier analysis demonstrated superior survival in the PPI group in comparison to patients who did not receive PPIs (log-rank test *p* = 0.032) (Figure 6).

The univariate and multivariate analyses for OS in esophageal cancer patients who received 5-FU-based chemotherapy revealed that PPI use was independently associated with OS (*p* = 0.009, HR, 0.33; 95% CI, 0.12–0.76) (Table 3).

There were no significant differences in the occurrence of grade ≥ 3 chemotherapy-related adverse events (Table 4). In the PPI group, two patients treated with docetaxel, cisplatin and 5-FU (DCF) developed grade 4 neutropenia, one of these patients developed febrile neutropenia. The other 5 patients (5-FU and cisplatin or 5-FU and nedaplatin) developed grade 3 neutropenia. In addition, grade 3 anorexia, grade 3 creatinine increase and grade 3 sinus bradycardia were observed in one case each. On the other hand, among the non-PPI group, 3 patients (2 5-FU and nedaplatin and 1 5-FU and cisplatin) had grade 4 neutropenia, and 5 patients had neutropenia. Three of these patients had febrile neutropenia. In addition, grade 3 anorexia and grade 3 creatinine increase were observed in one case each. Of the patients who experienced grade ≥ 3 chemotherapy-related adverse events, 8 were subsequently treated at a reduced intensity. In one case of grade 3 bradycardia and in one case of grade 3 creatinine increase, 5-FU and cisplatin was changed to 5-FU and nedaplatin. No cases were terminated due to side effects.

## 4. Discussion

In the present study, PPIs showed a dose-dependent antitumor effect on esophageal cancer cells and enhanced sensitivity to 5-FU at sublethal concentrations. In actual clinical practice, the oral PPI group showed a superior tumor response to 5-FU and better OS in comparison to the non-PPI group. These results indicate that PPI medication could enhance the chemosensitivity of esophageal cancer patients treated with 5-FU. To the best of our knowledge, this study is the first to demonstrate that PPIs could enhance the chemosensitivity of esophageal cancer both in vitro and in the clinical setting. Furthermore, the results in this study revealed, for the first time, that vonoprazan, a novel potassium-competitive acid blocker, has the effect of enhancing chemosensitivity.

The extracellular pH of solid tumors is more acidic in comparison to normal tissue as a consequence of high glycolysis and poor vascular perfusion [26]. Tumor cells have, thus, evolved the ability to function in a more acidic environment than normal cells. The activity of V-ATPase, a key pH regulator in tumor cells, is important for the excretion of excess acid into the extracellular environment, which results in a “reversed” pH gradient with a constitutively increased intracellular pH that is higher than the extracellular pH in tumor cells [27]. This reversal not only allows tumor cells to evade apoptosis, but is also involved in inducing drug resistance. At extracellular low pH, many drugs are protonated and lose their ability to enter cells, thus protecting the DNA of tumor cells from the effects of drugs [28,29]. In addition, the pH gradient between the cytoplasm and lysosomal compartment is also involved in resistance to the weakly basic chemotherapeutic drugs [30]. Therefore, the inhibition of V-ATPase by PPIs could alter pH regulation in tumor cells and overcome drug resistance. Consistent with previous reports [31], we confirmed that PPIs decreased the intracellular pH of esophageal cancer cell lines, suggesting that the increased sensitivity to 5FU induced by PPIs is mainly mediated by the changes in tumor cell pH regulation. However, in this study, the decrease in intracellular pH in lansoprazole-treated KYSE70 cells was weak, but the sensitivity to 5-FU was strongly enhanced in these cells, so there may be other mechanisms for enhancing the chemosensitivity of PPIs besides the change of intracellular pH. Further research is needed to elucidate the molecular mechanism by which PPIs exert enhanced chemosensitivity.

It has been reported that the rate of V-ATPase expression increases with the pathological grade and TNM stage in esophageal squamous cancer cells collected from patients [9]. It has also been reported that epithelial ovarian cancer patients whose mRNA expression of V-ATPase was in the upper 75th percentile showed significantly poorer overall survival in comparison to patients whose mRNA expression of V-ATPase was in the lower 25th percentile [22]. A number of these previous studies suggested that V-ATPase is a crucial factor that is involved in drug resistance, and tumor progression, and may represent a suitable and specific target for novel anticancer strategies. Pharmacologic inhibitors of V-ATPase activity have been used in the past with a high level of efficacy in vitro; however, their potential application in the clinical setting is hampered by predicted toxicity on normal cells [25,32]. Since the safety of PPIs is clinically well-established, the use of PPI as a V-ATPase inhibitor during chemotherapy is promising strategy for enhancing chemosensitivity. Recently, PPIs have been shown to enhance the clinical effect of chemotherapy in several cancer types [20,33]. For 5-FU-based chemotherapy, Wang et al. have shown to be better for OS (*p* = 0.04, RR, 0.72; 95% CI, 1.02–1.90) and PFS (*p* = 0.01, RR = 0.67; 95% CI, 1.10–2.05) in patients with colorectal cancer who were treated with a FOLFOX regimen along with PPIs than without PPIs [34]. In a cohort of 596 patients with previously untreated head and neck squamous cell carcinomas, PPI use was associated with improved OS (*p* < 0.0001, HR = 0.55; 95% CI, 0.42–0.73) [35]. Consistent with these reports, the analysis of our clinical data showed that OS in the oral PPI group was superior to that in the non-PPI group, and a multivariate analysis revealed that PPI use was independently associated with OS. Importantly, no additional toxicity was observed with PPI administration in the present study. Our results and recently published observations indicate a new path to anticancer therapy for drug-resistant tumors.

Although vonoprazan may be considered to be a PPI in a broad sense, it is a potassium-competitive acid blocker (P-CAB) with a different mechanism of action from lansoprazole and esomeprazole, which are PPIs in a narrow sense. A P-CAB was used as a PPI in this study because they are commonly and widely used in actual clinical practice as an alternative to PPIs due to their additional clinical advantages over conventional PPIs [36,37]. In the present study, vonoprazan showed a dose-dependent antitumor effect on esophageal cancer cell lines and enhanced sensitivity to 5-FU. This is the first study to evaluate the effect of vonoprazan on cancer cells, and these results suggest that vonoprazan is likely to function as a chemosensitizer in cancer treatment, similar to conventional PPIs. 

The present study was associated with several limitations. First, this was a single-center, retrospective study with a relatively small sample size. Second, in clinical practice, the criteria for administering PPIs were based on the judgment of the attending physician.

## 5. Conclusions

PPIs showed a dose-dependent antitumor effect on esophageal cancer, and sublethal concentrations of PPIs showed synergistic cytotoxicity with 5-FU in vitro. In actual clinical practice for patients treated with 5-FU for advanced esophageal cancer, the OS of the oral PPI group was superior to that of the non-PPI group, and the occurrence of chemotherapy-related adverse events in the two groups did not differ to a statistically significant extent. Although this was a small-sized retrospective study, these results suggest that the use of PPIs could safely enhance chemosensitivity in patients with esophageal cancer. The effect of PPIs as a chemosensitizer in patients with esophageal cancer needs to be investigated in a large prospective controlled study and the underlying molecular mechanisms also need to be further studied in the future.

## Figures and Tables

**Table 1:** Baseline characteristics of esophageal cancer patients taking PPIs vs. esophageal cancer patients not taking PPIs.

Characteristics			PPI (*n* = 18)	Non-PPI (*n* = 22)	*p* Value
Age, mean (SD), years			67.9 (7.83)	65.6 (7.90)	0.364
Sex, n					
	Male		16	17	0.328
	Female		2	5	
PS, n					
	0		6	11	0.264
	1		12	10	
	2		0	1	
BMI, mean (SD), kg/m^2^			21.2 (3.90)	19.4 (3.12)	0.112
Location, n					
	U		2	4	0.490
	M		7	11	
	L		9	7	
Disease status, n					
	Metastatic		17	18	0.212
	Recurrent		1	4	
Prognostic factors					
	CCI, n				
	0		9	14	0.596
	1–2		7	7	
	≥3		2	1	
	GPS, n				
	0		9	11	0.548
	1		7	6	
	2		2	5	
	PNI, mean (SD)		44.8 (4.23)	43.6 (7.53)	0.529
	NLR, mean (SD)		3.6 (2.32)	5.2 (7.84)	0.409
Regimen (1st course), n					
	FP		11	16	0.500
	DCF		5	4	
	Nedaplatin FU		1	2	
	FOLFOX		1	0	
Radiation therapy, n					
	Yes		13	11	0.150
	No		5	11	
PPI subtypes, daily dose, n					
	Lansoprazole			-	-
		15 mg	2		
		30 mg	6		
	Rabeprazole				
		10 mg	3		
	Esomeprazole				
		20 mg	4		
	Vonoprazan				
		10 mg	1		
		20 mg	2		

PS, performance status; BMI, body mass index; CCI, Charlson comorbidity index; GPS, Glasgow prognostic score; PNI, prognostic nutritional index; NLR, neutrophil lymphocyte ratio; FP, fluorinated pyrimidines; DCF, docetaxel plus 5-fluorouracil and cisplatin; Nedaplatin FU, Nedaplatin and 5-fluorouracil; FOLFOX, oxaliplatin plus fluorouracil and leucovorin.

**Table 2:** Clinical outcomes of esophageal cancer patients in the PPI and non-PPI groups.

Response to Treatment	PPI (*n* = 18)	Non-PPI (*n* = 22)	Treatment Difference (95% CI)	*p* Value
CR, *n* (%)	0	1 (4.5)		0.269
PR, *n* (%)	12 (66.7)	8 (36.4)		
SD, *n* (%)	5 (27.8)	8 (36.4)		
PD, *n* (%)	1 (5.6)	5 (22.7)		
Response rate, *n* (%)	12 (66.7)	9 (40.9)	0.35 (0.09–1.27)	0.102
Disease control rate, *n* (%)	17 (94.4)	17 (77.3)	0.2 (0.02–1.90)	0.113

CR, complete response; PR, partial response; SD, stable disease; PD, progressive disease.

**Table 3:** Univariate and multivariate analyses of risk factors for overall survival in esophageal cancer patients treated with 5-FU-based chemotherapy.

	Univariate Analysis			Multivariate Analysis		
	HR	95% CI	*p* Value	HR	95% CI	*p* Value
Age						
<75 years	1					
≥75 years	0.70	0.20–1.84	0.496			
Sex						
Female	1					
Male	2.05	0.78–7.07	0.161			
PS						
=0	1			1		
≥1	1.20	0.56–2.59	0.642	1.32	0.53–3.30	0.549
Regimen (1st course)						
DCF	1					
Other	0.91	0.42–2.15	0.825			
CCI						
0	1					
≥1	1.38	0.65–2.95	0.408			
GPS						
=0–1	1			1		
=2	2.32	0.76–5.90	0.130	2.59	0.68–8.88	0.154
PNI						
≥45	1					
<45	1.18	0.55–2.59	0.670			
NLR						
<5	1			1		
≥5	1.93	0.74–4.50	0.169	1.88	0.64–5.08	0.236
PPI						
No	1			1		
Yes	0.41	0.17–0.92	0.029	0.35	0.13–0.80	0.012

HR, hazard ratio; CI, confidence interval; PS, performance status; CCI, Charlson comorbidity index; GPS, Glasgow prognostic score; PNI, prognostic nutritional index; NLR, neutrophil lymphocyte ratio.

**Table 4:** Chemotherapy-related adverse events (grade ≥ 3) for esophageal cancer patients taking PPIs vs. patients without PPI treatment.

	PPI (*n* = 18)	Non-PPI (*n* = 22)	Odds Ratio (95% CI)	*p* Value
Myelosuppression, *n*	7	9	0.92 (0.26–3.28)	0.897
Gastrointestinal toxicity, *n*	1	1	1.24 (0.07–21.2)	0.884
Heart failure, *n*	1	0	-	0.202
Renal toxicity, *n*	1	2	0.59 (0.05–7.07)	0.669

## Data Availability

Data will be available from the corresponding author upon reasonable request.

## Supplementary Materials

The following supporting information can be downloaded at: https://www.mdpi.com/article/10.3390/cancers14102395/s1, Figure S1: Whole western blot and densitometry readings/intensity ratio of V-ATPase C1 in the KYSE50 and KYSE70 cell lines; Table S1: The combination index (CI) was calculated by Compusyn software, and a CI value < 1 represents synergism.
