# Supplementary material for: Proton Pump Inhibitors Enhance the Antitumor Effect of Chemotherapy for Esophageal Squamous Cell Carcinoma
Source: Cancers (Basel). 2022 May 12;14(10):2395. doi: 10.3390/cancers14102395 (PMC9140098; doi:10.3390/cancers14102395)
Supplement: Supplementary file 1 [file cancers-14-02395-s001.zip › cancers-1697243-supplementary.pdf]

cancers

MDPI

Article

Proton Pump Inhibitors Enhance the Antitumor Effect of Chemotherapy for Esophageal Squamous Cell Carcinoma

Shinya Matsumura [1,2], Takeshi Ishikawa [1,*], Juichiro Yoshida [1], Ryuichi Morita [1], Tomoki Sakakida [1], Yuki Endo [1], Toshifumi Doi [1], Ryohei Hirose [1], Ken Inoue [1], Osamu Dohi [1], Naohisa Yoshida [1], Kazuhiko Uchiyama [1], Tomohisa Takagi [1], Hideyuki Konishi [1], Kohichiroh Yasui [3], Yuji Naito [1] and Yoshito Itoh [1]

[1] Department of Molecular Gastroenterology and Hepatology, Graduate School of Medical Science, Kyoto Prefectural University of Medicine, Kyoto 602-8566, Japan; matsumu@koto.kpu-m.ac.jp (S.M.); jyoshida@koto.kpu-m.ac.jp (J.Y.); mryuich@koto.kpu-m.ac.jp (R.M.); stomoki@koto.kpu-m.ac.jp (T.S.); endo0622@koto.kpu-m.ac.jp (Y.E.); t-doi@koto.kpu-m.ac.jp (T.D.); ryo-hiro@koto.kpu-m.ac.jp (R.H.); keninoue71@koto.kpu-m.ac.jp (K.I.); osamu-d@koto.kpu-m.ac.jp (O.D.); naohisa@koto.kpu-m.ac.jp (N.Y.); k-uchi@koto.kpu-m.ac.jp (K.U.); takatomo@koto.kpu-m.ac.jp (T.T.); hkonishi@koto.kpu-m.ac.jp (H.K.); ynaito@koto.kpu-m.ac.jp (Y.N.); yitoh@koto.kpu-m.ac.jp (Y.I.)

[2] Department of Gastroenterology, Kyoto Chubu Medical Center, Kyoto 629-0197, Japan

[3] Department of Nursing, Faculty of Health Sciences, Bukkyo University, Kyoto 603-8301, Japan; yasuik@koto.kpu-m.ac.jp

* Correspondence: iskw-t@koto.kpu-m.ac.jp; Tel.: +81-75-251-5519; Fax: +81-75-251-0710

Table S1. The combination index (CI) was calculated by Compusyn software, and a CI value < 1 represents synergism.

KYSE50			
**Concentration of 5-FU (μM)**	**Concentration of lansoprazole (μM)**	**Cell viability rate**	CI
2	25	0.696	0.638
20	25	0.551	0.383
200	25	0.474	0.34
	Concentration of esomeprazole (μM)		
2	10	0.784	0.442
20	10	0.602	0.243
200	10	0.528	0.37
	Concentration of vonoprazane (μM)		
2	10	0.777	0.321
20	10	0.686	0.276
200	10	0.572	0.424
KYSE70			
Concentration of 5-FU (μM)	Concentration of lansoprazole (μM)	Cell viability rate	CI
2	5	0.765	0.299
20	5	0.485	0.166
200	5	0.364	0.605
	Concentration of esomeprazole (μM)		
2	5	0.7	0.644
20	5	0.31	0.368
200	5	0.147	0.79
	Concentration of vonoprazane (μM)		
2	50	0.821	1.776
20	50	0.572	0.585
200	50	0.448	0.584

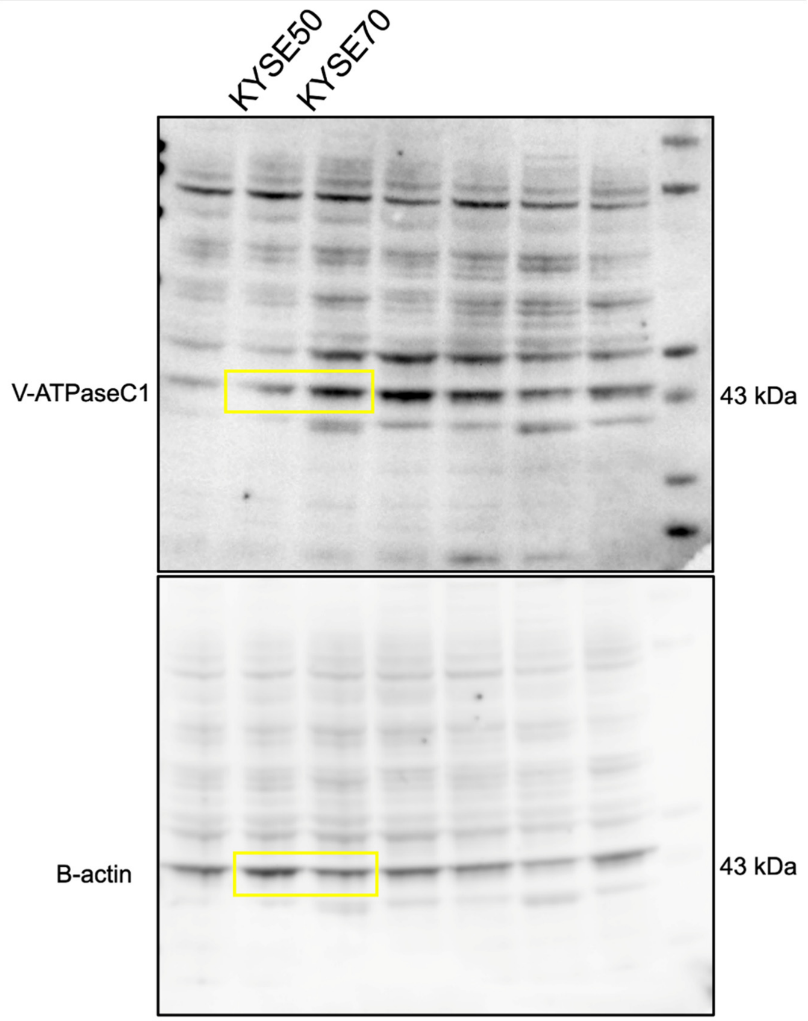

	KYSE50	KYSE70
V-ATPaseC1/B-actin	0.936	1.517

Figure S1. Whole western blot and densitometry readings/intensity ratio of V-ATPase C1 in the KYSE50 and KYSE70 cell lines.
